# High-quality haplotype-resolved genome assembly and annotation of *Malus baccata* ‘Jackii’

**DOI:** 10.1038/s41597-025-06504-5

**Published:** 2026-01-08

**Authors:** Matthias Pfeifer, Ofere Francis Emeriewen, Henryk Flachowsky, Monika Höfer, Jens Keilwagen, Fang-Shiang Lim, Andreas Peil, Holger Zetzsche, Thomas Wöhner

**Affiliations:** 1 Julius Kühn-Institut (JKI) - Federal Research Centre for Cultivated Plants, Institute for Breeding Research on Fruit Crops, Dresden-Pillnitz, Germany; 2 Institute of Plant Genetics, Department of Molecular Plant Breeding, Leibniz University Hannover, Hannover, Germany; 3 Julius Kühn-Institut (JKI) - Federal Research Centre for Cultivated Plants, Institute for Biosafety in Plant Biotechnology, Quedlinburg, Germany; 4 Julius Kühn-Institut (JKI) - Federal Research Centre for Cultivated Plants, Institute for Resistance Research and Stress Tolerance, Quedlinburg, Germany

**Keywords:** Genome, Plant breeding

## Abstract

*Malus baccata* ‘Jackii’ has been observed to exhibit multiple disease resistances, thus rendering it a promising source for breeding new disease-resistant apple cultivars. Here, we present the first haplotype-resolved genome assembly and annotation of this genotype, achieved by integrating PacBio HiFi sequencing, Hi-C, and mRNA sequencing data with a range of bioinformatic tools and databases. The genome assembly comprises 17 pseudochromosomes with total scaffold lengths of 654.6 Mb and 637.5 Mb for the two haplotypes, respectively. Both haplotypes have scaffold N50 values exceeding 30 Mb, with 42,441 and 46,507 predicted genes, of which 99.9% were successfully annotated. The high quality of this genome is supported by BUSCO analysis values exceeding 97.5% for both haplotypes. This comprehensive dataset is well suited for a wide range of future genomic analyses and is anticipated to benefit apple breeding, particularly in the context of enhancing disease resistance.

## Background & Summary

The apple (*Malus domestica* Borkh.) is among the most popular and important fruits in the world. Different closely related species of apple (*Malus* spp.) can hybridize easily, and the domesticated apple of today contains genomic contributions from e.g. *Malus sieversii*, *Malus sylvestris*, *Malus orientalis* and *Malus baccata*^1^. *M. baccata* is native to Asia and is used for breeding cultivars and rootstocks due to its distinct cold hardiness and disease resistance^2^. Breeding of new apple varieties is a complex process that takes several years^3^. Sequencing the genomes of apple genotypes provides insights into genetic diversity, evolutionary history, and genotype-phenotype relationships. The availability of whole genome sequences facilitates the development of marker-assisted selection (MAS), which aids breeding by enabling more efficient and targeted selection strategies. Recently, many genomes from various apple cultivars and individual accessions of different *Malus* species have been sequenced and published^4^, including that of *M. baccata*^2^. However, until now, no genome sequence has been available for *M. baccata* ‘Jackii’, an ornamental genotype collected in 1905 by J. G. Jack in Seoul^5^, which exhibits resistance to several fungal diseases such as apple scab (*Venturia inaequalis*)^6^, powdery mildew (*Podosphaera leucotricha*)^7^ and apple blotch (*Diplocarpon coronariae)*^8^. In addition, *M. baccata* ‘Jackii’ is resistant to fire blight^9,10^, caused by the Gram-negative bacterium *Erwinia amylovora*, which is one of the most destructive bacterial diseases affecting the genus *Malus*^11^. Breeding resistant apple cultivars is a promising and desirable strategy against fire blight and would require pyramiding the different resistance gene (R-gene) candidates due to the fact that resistance is strain-dependent, with some R-gene donors already overcome by virulent strains of *E. amylovora*^11,12^. The first fire blight R-gene to be isolated and functionally characterized using a transgenic approach was *FB_Mr5*, which underlies the resistance QTL region at the top of chromosome 3 of *Malus × robusta* 5^13,14^. *FB_Mr5* encodes a CC-NBS-LRR resistance protein that interacts with the cysteine protease AvrRpt2_*Ea*_ from *E. amylovora*, demonstrating a gene-for-gene relationship^9,13–16^. Moreover, sequence analysis of *avrRpt2*_*Ea*_ from various *E. amylovora* strains, along with inoculation experiments using *avrRpt2*_*Ea*_ knock-out mutants, revealed that *FB_Mr5*-mediated resistance in *Malus × robusta* 5 is lost when inoculated with knockout mutants and strains carrying a cysteine-to-serine substitution at amino acid position 156 in the bacterial effector *avrRpt2*_*Ea*_. Furthermore, it was shown that fire blight resistance in *M. baccata* ‘Jackii’ likely functions in a similar manner to that of *Malus × robusta* 5^9,16^. Additionally, a closely related homologue of *FB_Mr5* was identified in *M. baccata* ‘Jackii’^17^. However, as it has been demonstrated that even a single amino acid substitution in specific regions of FB_Mr5 can have a significant impact and trigger autoactivity^18^, it is important to know the exact sequences of resistance genes and whether there are other candidate genes at an R-gene locus that show a very high sequence similarity. In this study, we generated a high-quality, haplotype-resolved genome assembly and annotation of *M. baccata* ‘Jackii’ by combining PacBio HiFi sequencing with Hi-C and mRNA sequencing. Tunable genotyping-by-sequencing (tGBS)^19^ data of an F_1_ biparental population derived from an ‘Idared’ × *M. baccata* ‘Jackii’ cross were generated, and single-nucleotide polymorphisms (SNPs) were identified by mapping the sequences to the newly assembled genome. By additionally mapping the sequences of the tGBS analysis to the HFTH1 reference genome, chromosome names were assigned based on the HFTH1 assembly^20^. The genome assembly and annotation presented here for this multi-resistant genotype are of significant value and can be utilised directly for various resistance analyses and other applications.

## Methods

### Sampling, DNA and RNA extraction

Leaves from *M. baccata* ‘Jackii’ were collected at the Fruit Genebank of the Julius Kühn-Institut (JKI) in Dresden-Pillnitz, Germany. The QIAGEN Genomic-tip 20/G kit (QIAGEN, Hilden, Germany) was used for DNA extraction, while the RNAprep Pure Plant Plus Kit (Tiangen, Beijing, China) was employed for RNA extraction, with both procedures conducted in accordance with the manufacturer’s protocols.

### Genome size estimation using Illumina sequencing

**Fig. 1:**
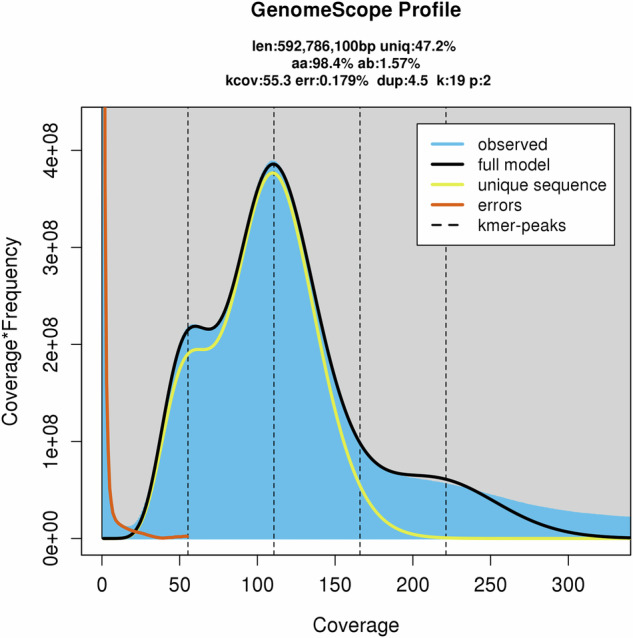
K-mer distribution map (k = 19) of *Malus baccata* ‘Jackii’.

Genomic DNA from diploid *M. baccata* ‘Jackii’ was sequenced using the Illumina NovaSeq. 6000 platform (Illumina, Inc., San Diego, CA, USA) with a paired-end read length of 150 bp and a 350 bp sequencing library. Subsequently, the reads were quality-filtered (polyG tails trimmed, minimum length ≥ 100 bp, average read quality ≥ Q20, homopolymer filter ≤ 10% consecutive identical bases, ≤50% of bases with Q < 10). After filtering, a total of 80.14 GB of sequencing data was obtained, corresponding to an estimated sequencing depth of ~135.19 × . The GC content was 37.22%, with Q20 exceeding 97.85% and Q30 surpassing 93.87%. Genome analysis was conducted using the Jellyfish 2.1.4^21^ and GenomeScope 2.0^22^ software. The k-mer distribution map with k = 19 was generated, the haploid genome size of *M. baccata* was estimated to be 592.8 Mb and the heterozygosity rate was calculated to be ~1.57% (Fig. 1). The repeat sequence content was estimated at ~52.78%.

### Haplotype-resolved genome assembly with PacBio and Hi-C

DNA from *M. baccata* ‘Jackii’ was used for PacBio HiFi sequencing on the PacBio Revio platform (Pacific Biosciences, Menlo Park, CA, USA) following the standard protocol. The DNA molecules were sequenced in zero-mode waveguides (ZMWs) over multiple cycles, and repeated subreads were combined to generate highly accurate, self-corrected HiFi reads. In total, 6,149,970 HiFi reads were produced, yielding 101.3 Gb of sequence data. The average HiFi read length was 16,479 bp, with an N50 of 16,808 bp, and the longest HiFi read measured 61,744 bp.

In addition, an *in situ* Hi-C experiment was conducted^23^. To preserve DNA-DNA interactions and maintain the 3D genome structure, cross-linking was performed with formaldehyde and subsequently DNA was digested with the *Hin*dIII restriction enzyme generating sticky ends that were filled in with biotin-labeled nucleotides. Blunt-end ligation was performed to form circular structures. After reversing the cross-linking, the DNA was purified and sheared into fragments between 300–700 bp. Biotinylated junctions were isolated using streptavidin beads, and purified fragments were utilised for library preparation and sequencing on an Illumina NovaSeq. 6000 PE150 (Illumina, Inc., San Diego, CA, USA). This process generated 689.2 M read pairs, corresponding to 206.3 Gb of sequence data, with an average GC content of 39.54%, a Q20 value of 97.98%, and a Q30 value of 94.70%. The Hi-C data were processed using HiC-Pro v2.10.0^24^, and the paired-end reads were aligned using BWA (v0.7.10-r789; mode: aln; default settings)^25^ to the preliminary assemblies of haplotype 1 and haplotype 2. These preliminary haplotype-resolved assemblies were generated using only HiFi reads with Hifiasm (v0.19.9-r616)^26^ through three main steps: haplotype-aware error correction, which preserves heterozygous sites to maintain phasing accuracy, phased string-graph construction, and contig generation. Of the total 1,378.4 M Hi-C reads, 1,174.7 M and 1,175.4 M reads were mapped to the preliminary assemblies of haplotype 1 and 2, respectively. Among these, 538.1 M and 538.9 M reads were uniquely mapped, resulting in 196.9 M (36.59%) and 196.6 M (36.48%) valid interaction pairs for haplotype 1 and 2, respectively. The preliminary assembly was segmented into 50-kb fragments and scaffolded into haplotype-resolved pseudochromosomes using Hi-C data with LACHESIS^27^. The following optimised parameters were applied: CLUSTER_MIN_RE_SITES = 118, CLUSTER_MAX_LINK_DENSITY = 2, ORDER_MIN_N_RES_IN_TRUNK = 75 (haplotype 1) / 15 (haplotype 2), and ORDER_MIN_N_RES_IN_SHREDS = 87 (haplotype 1) / 15 (haplotype 2). In total, 98.1% and 99.0% of the Hi-C-derived sequences were anchored for haplotypes 1 and 2, respectively, with 97.3% and 98.8% confirmed in correct order and orientation. A summary of the Hi-C-based haplotype-resolved genome assembly is presented in Table 1.

**Table 1:** Summary of the Hi-C-based haplotype-resolved genome assembly (only scaffolds >1 kb were included).

Haplotype	1	2
**Number of scaffolds**	719	272
**Total length of scaffolds (Mb)**	654.6	637.6
**Scaffold N50 length (Mb)**	35.8	36.6
**Scaffold N90 length (Mb)**	30.2	30.1
**Maximum length of scaffold (Mb)**	51.8	51.8
**Number of contigs**	740	292
**Total length of contigs (Mb)**	654.6	637.6
**Contig N50 length (Mb)**	30.4	30.7
**Contig N90 length (Mb)**	9.9	11.5
**Maximum length of the contigs (Mb)**	51.8	51.8
**GC content (%)**	38.14	38.02
**Gap numbers**	21	20

**Fig. 2:**
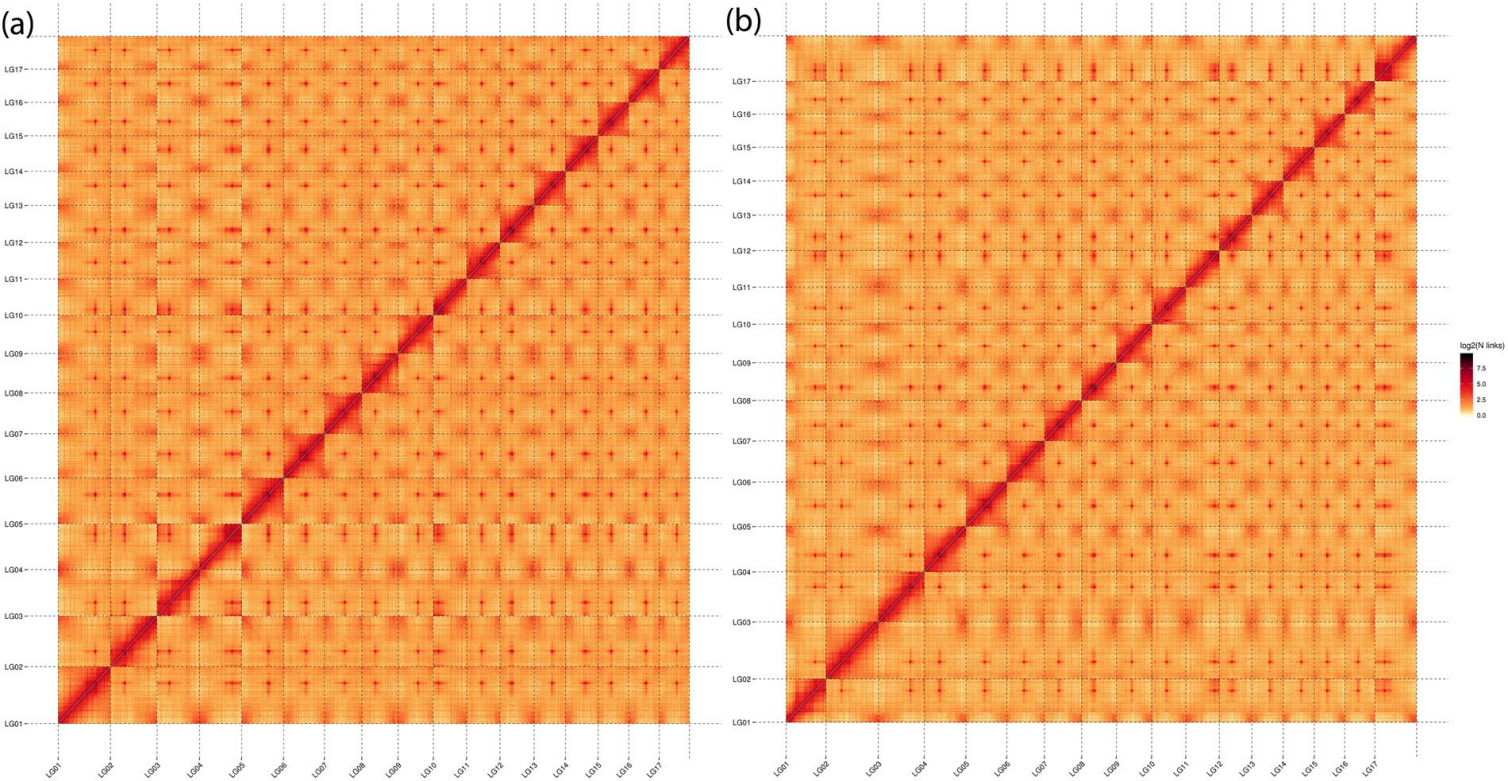
Heatmap of the assembled haplotype-resolved genomes. (**a**) Haplotype 1. (**b**) Haplotype 2.

The assembled genome sequence was cut into 300 kb bins, and the signal intensity between corresponding bins was visualized as a heatmap in Fig. 2. The signal intensity was stronger within the 17 chromosome groups than between them, indicating a high-quality genome assembly.

### Transcriptome sequencing and genome annotation

The assembled genome was then used for genome annotation, with transposable element prediction performed using the following programs and databases: RepeatModeler2 v2.0.1^28^, RECON v1.0.8^29^, RepeatScout v1.0.6^30^, LTR_retriever v2.8^31^, LTRharvest v1.5.9^32^, LTR_FINDER v1.1^33^, RepeatMasker v4.1.0^34^, Repbase v19.06^35^, REXdb v3.0^36^ and Dfam v3.2^37^. Tandem repeats were predicted using the Microsatellite identification tool (MISA v2.1)^38^ and the Tandem Repeat Finder (TRF, v409)^39^. The results of the aforementioned analyses are presented in Tables 2 and 3.

**Table 2:** Summary of predicted transposable elements.

Type of transposable element	Number of predicted repetitive sequences (HT1/HT2)	Sequence length in bp (HT1/HT2)	Percentage of genome (HT1/HT2)
Class I: DIRS	2/3	122/169	0.00/0.00
Class I: LINE	33,232/29,751	19,082,791/12,588,448	2.92/1.97
Class I: LTR Caulimovirus	1,167/1,511	1,680,429/1,567,889	0.26/0.25
Class I: LTR Copia	74,041/75,763	61,731,539/60,267,561	9.43/9.45
Class I: LTR ERV	4,421/4,361	404,792/335,617	0.06/0.05
Class I: LTR Gypsy	84,129/81,591	102,424,853/99,955,957	15.65/15.68
Class I: LTR Ngaro	285/300	21,356/23,580	0.00/0.00
Class I: LTR Pao	135/93	12,134/6,123	0.00/0.00
Class I: LTR unknown	176,052/180,631	78,416,294/81,106,872	11.98/12.72
Class I: SINE	16,693/16,433	2,778,885/2,248,211	0.42/0.35
Total for Class I	390,157/390,437	266,553,195/258,100,427	40.72/40.48
Class II: Academ	1/1	138/138	0.00/0.00
Class II: CACTA	2,613/3,019	1,102,211/983,375	0.17/0.15
Class II: Crypton	22/34	1,050/1,759	0.00/0.00
Class II: Dada	243/233	12,084/11,497	0.00/0.00
Class II: Ginger	35/33	1,712/1,407	0.00/0.00
Class II: Helitron	147,768/141,516	37,977,788/42,416,743	5.80/6.65
Class II: IS3EU	179/187	10,934/10,119	0.00/0.00
Class II: Kolobok	276/290	20,418/23,008	0.00/0.00
Class II: Maverick	156/241	13,930/22,666	0.00/0.00
Class II: Merlin	149/140	7,271/7,513	0.00/0.00
Class II: Mutator	461/541	29,174/38,009	0.00/0.01
Class II: P	75/81	5,351/4,763	0.00/0.00
Class II: PIF-Harbinger	5,776/5,750	3,113,051/3,104,728	0.48/0.49
Class II: PiggyBac	66/66	3,304/2,620	0.00/0.00
Class II: Sola	0/1	0/95	0.00/0.00
Class II: Tc1-Mariner	212/225	12,471/13,478	0.00/0.00
Class II: unknown	126,862/121,450	30,761,945/28,104,547	4.70/4.41
Class II: Zisupton	95/100	4,301/4,596	0.00/0.00
Class II: hAT	4,953/4,985	2,128,608/2,120,043	0.33/0.33
Total for Class II	289,942/278,893	75,205,741/76,871,104	11.49/12.06
Unknown	39/28	2,455/1,492	0.00/0.00
Total	680,138/669,358	341,761,391/334,973,023	52.21/52.54

HT: haplotype.

**Table 3:** Summary of tandem repeat analysis.

Type of tandem repeat	Number of predicted repetitive sequences (HT1/HT2)	Sequence length in Mb (HT1/HT2)	Percentage of genome (HT1/HT2)
Microsatellite (1–9 bp units)	262,351/257,143	4.7/4.7	0.72/0.73
Minisatellite (10–99 bp units)	166,504/161,310	15.9/15.3	2.42/2.40
Satellite (≥100 bp units)	24,351/23,003	12.4/11.6	1.90/1.82
Total	453,206/441,456	33.0/31.5	5.04/4.95

HT: haplotype.

**Fig. 3:**
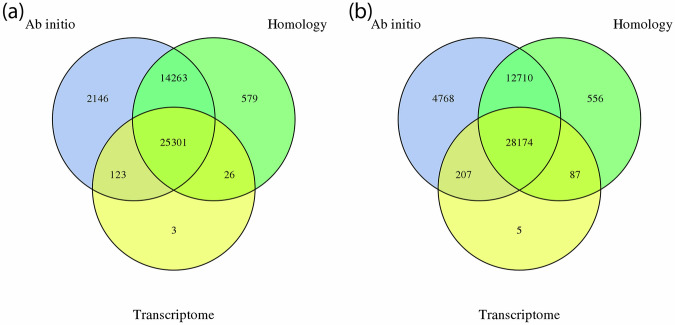
Venn diagram of integrated predicted coding genes. (**a**) Haplotype 1. (**b**) Haplotype 2. Created with Venny^81^.

The coding gene prediction was performed using three complementary approaches: *ab initio*, homology-based, and transcriptome-based methods (with and without reference genomes). *Ab initio* coding gene prediction was carried out using Augustus v2.4^40^ and SNAP (2006-07-28)^41^. Homology-based predictions were performed with GeMoMa v1.7^42^, and for transcriptome-based predictions, mRNA sequencing was performed on Illumina Novaseq. 6000 platform (Illumina, Inc., San Diego, CA, USA) with a paired-end read length of 150 bp. This yielded a total of 40.3 M reads, corresponding to 12.1 Gb. The Q30 and Q20 values were 94.57% and 98.01%, respectively, and the GC content was 48.16%. Transcripts were predicted using HISAT v2.0.4^43^ and StringTie v1.2.3^44^ with different reference genomes^20,45–47^. Coding genes were then identified using GeneMarkS-T v5.1^48^. Additionally, transcripts were assembled without the use of a reference genome with Trinity v2.11^49^, and coding genes were predicted with PASA v2.0.2^50^. Finally, the genes predicted by the different methods were integrated using EVM v1.1.1^51^ and finalized by PASA v2.0.2^50^. The total number of coding genes predicted for haplotype 1 and 2 was 42,441 and 46,507, respectively. Detailed results are presented in Tables 4 and 5 and in Fig. 3.

**Table 4:** Predicted coding genes using different software tools.

Prediction method	Gene prediction software	Used reference genome	Predicted genes (HT1/HT2)
*Ab initio*	Augustus		43,936/38,861
*Ab initio*	SNAP		71,540/67,191
Homology-based	GeMoMa	*A. thaliana*	35,054/34,405
Homology-based	GeMoMa	*M. domestica* HFTH1	59,817/58,640
Homology-based	GeMoMa	*M. domestica* GDDH13	46,953/46,238
Homology-based	GeMoMa	*M. prunifolia*	45,006/44,218
Transcriptome-based	GeneMarkS-T		25,498/25,141
Transcriptome-based	PASA		25,107/24,843
Integration/Finalization	EVM/PASA		42,441/46,507

HT: haplotype.

**Table 5:** Statistics of predicted coding genes of *Malus baccata* ‘Jackii’.

Haplotype	1	2
**Number of predicted genes**	42,441	46,507
**Total length of genes (Mb)**	156.5	157.3
**Average gene length**	3,687.96	3,382.22
**Total length of exons (Mb)**	69.6	71.2
**Number of exons**	234,373	238,393
**Average exons per gene**	5.52	5.13
**Total length of CDS (Mb)**	59.0	60.7
**Total length of introns (Mb)**	86.9	86.1
**Number of introns**	191,932	191,886
**Average introns per gene**	4.52	4.13

CDS: coding DNA sequence

Non-coding RNA prediction was conducted with tRNAscan-SE v1.3.1^52^ for tRNA, with barrnap v0.9^53^ based on Rfam v12.0^54^ for rRNA, with miRBase^55^ for miRNA, and with Infernal 1.1^56^ based on Rfam v12.0^54^ for snoRNA and snRNA. The results are summarised in Table 6.

**Table 6:** Predicted numbers of non-coding RNAs.

Haplotype	rRNA	tRNA	miRNA	snRNA	snoRNA
**1**	7,209	1,400	120	96	114
**2**	5,102	933	119	92	96

Homologous gene sequences without a complete gene locus were identified using GenBlastA v1.0.4^57^ and GeneWise v2.4.1^58^ was used to detect premature stop codons and frameshift mutations, resulting in the identification of 228 and 308 pseudogenes, respectively (Table 7).

**Table 7:** Predicted pseudogenes in *Malus baccata* ‘Jackii’.

Haplotype	Number of predicted pseudogenes	Total length of predicted pseudogenes (bp)	Average length of pseudogenes (bp)
**1**	228	975,909	4,280.30
**2**	308	1,282,971	4,165.49

The predicted coding genes were functionally annotated using multiple databases, including GenBank Non-Redundant (NR, 20200921), eggNOG 5.0^59^, Gene Ontology (GO, 20200615)^60,61^, Kyoto Encyclopedia of Genes and Genomes (KEGG, 20191220)^62^, SWISS-PROT and TrEMBL (202005)^63^, Pfam v33^64^ and eukaryotic orthologous groups (KOG, 20110125). Overall, more than 99.9% of coding genes were successfully annotated. The statistics on gene function annotation are presented in Table 8.

**Table 8:** Statistics of gene function annotation.

Database	Number of annotated genes (HT1/HT2)	Percentage of annotated genes (HT1/HT2)
GO	33,072/33,956	77.92/73.01
KEGG	30,493/31,763	71.85/68.3
KOG	22,503/23,325	53.02/50.15
Pfam	34,025/35,128	80.17/75.53
SWISS-PROT	30,036/30,202	70.77/64.94
TrEMBL	42,402/46,449	99.91/99.88
eggNOG	34,189/35,057	80.56/75.38
NR	41,015/44,867	96.64/96.47
Total	42,408/46,461	99.92/99.9

HT: haplotype.

In the final step, InterProScan (5.34-73.0)^65^ was utilised for the prediction of motifs and domains. A total of 1,876 motifs and 45,003 domains were predicted in haplotype 1, and 1,820 motifs and 44,973 domains in haplotype 2.

### Chromosome assignment according to HFTH1

**Fig. 4:**
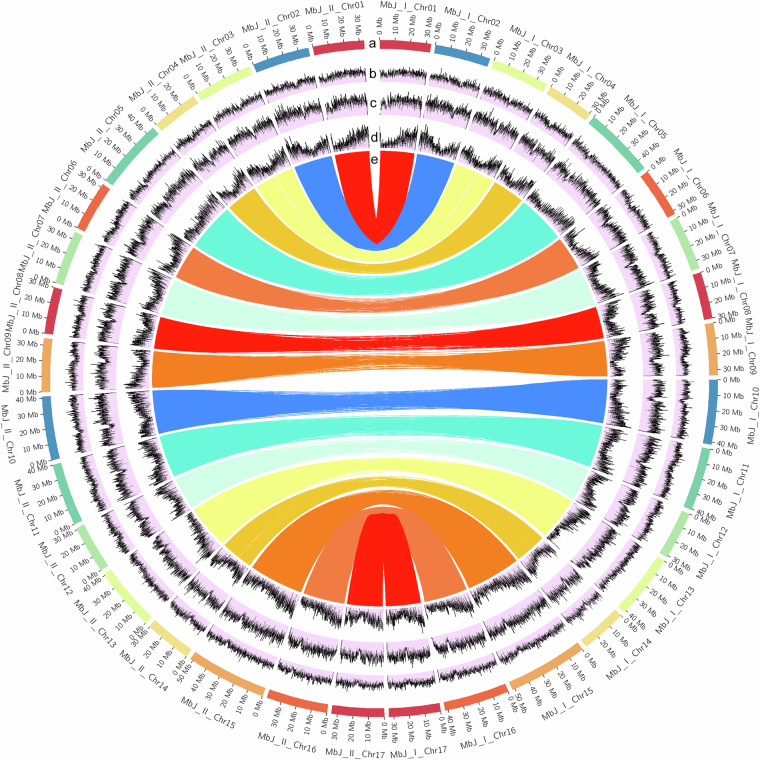
Circos plot of the *Malus baccata* ‘Jackii’ haplotype-resolved genome assembly. (**a**) Chromosome names and lengths in Mb. (**b**) Frequency of tandem repeats in 50 kb windows. (**c**) Frequency of transposable elements in 50 kb windows. (**d**) Frequency of genes in 50 kb windows. (**e**) Sequences from the tGBS analysis of haplotype 1 mapped onto haplotype 2. kb: kilobase, Mb: million base pairs, tGBS: tunable genotyping-by-sequencing.

DNA of 184 F_1_ individuals from the cross ‘Idared’ × *M. baccata* ‘Jackii’, including the parental genotypes, was analysed using tGBS^19^ by Data2Bio (Ames, IA, USA). The restriction enzyme *Bsp*1286I was used and sequencing was carried out on an Illumina HiSeq X instrument (Illumina, Inc., San Diego, CA, USA). Polymorphic sites were first identified, and in a second step, final SNP calling was performed. In the initial step, individual sequence reads were scanned for low-quality regions (PHRED score ≤15), and quality-trimmed sequence reads were aligned to the genome sequences of haplotypes 1 and 2 of *M. baccata* ‘Jackii’ reported here using GSNAP^66^. Only confidently mapped reads with ≤2 mismatches per 36 bp and no more than 4 bases as tails per 75 bp that aligned to a single location were used for SNP identification, based on the following criteria: for homozygous SNPs, the most common allele had to be supported by at least 5 unique reads and 80% of all aligned reads. For heterozygous SNPs, each of the two most common alleles had to be supported by at least 5 unique reads and at least 30% of all aligned reads. For both homozygous and heterozygous SNPs, polymorphisms in the first and last 3 bp of each quality-trimmed read were ignored, and a PHRED base quality value of 20 (≤1% error rate) was set as the threshold for each polymorphic base. In the second step, SNPs were classified for tGBS genotyping as follows: a SNP was classified as homozygous if ≥5 reads supported the major allele and ≥90% of all reads at that site matched, and heterozygous if ≥2 reads supported each of two alleles, both alleles individually made up >20%, and their combined reads were ≥5, covering ≥90% of all reads at that site. SNPs were then filtered based on a minimum calling rate of 50%, the allele number was set to 2, the number of genotypes ≥2, the minor allele frequency ≥10%, and the heterozygosity rate range between 0% and (2 × Frequency_allele1_ × Frequency_allele2_ + 20%). In a final step, imputation was used on chromosome-based SNPs that lacked a sufficient number of reads to make genotype calls using Beagle v5.4^67^ with 50 phasing iterations and default parameters. A total of 321,733 and 319,620 SNPs for haplotypes 1 and 2 were identified, with each site genotyped in at least 50% of the samples. SNP sequences from the tGBS data were then mapped to the HFTH1 genome sequence^20^ using BWA-MEM2^68^ on the JKI Galaxy Server (Galaxy v2.2.1 + galaxy1)^69^ and the data presented in this study were assigned and oriented according to the HFTH1 reference^20^. A Circos plot^70^ illustrating key genomic features and alignments between the two haplotypes is shown in Fig. 4.

## Data Records

The raw data and assembled sequences and annotations can be accessed from the European Nucleotide Archive (ENA) under the BioProject accession number PRJEB89942^71^ and study number ERP172974. The SNPs and their corresponding sequence data, as well as the adjusted genome sequences (Mbj_HT1/Mbj_HT2.fasta), gene and peptide sequences (cds.fasta and pep.fasta), and annotation data (final tandem repeats, TE final, EVM.final.gene, domains and motifs) are available from Figshare^72^.

## Technical Validation

### BUSCO analysis

To assess the completeness of the genome assembly, a BUSCO analysis with BUSCO v4.0^73^ was performed using the Embryophyta database containing 1,614 core genes. The results, presented in Table 9, demonstrate the high integrity of the haplotype-resolved genome assembly, with 97.58% and 97.52% of the core genes identified in haplotype 1 and 2, respectively.

**Table 9:** BUSCO analysis results using the Embryophyta database (1,614 core genes).

Haplotype	Total number of identified core genes	Number of identified single-copy core genes	Number of identified duplicated core genes	Number of identified fragmented core genes	Number of missing core genes
**1**	1,575 (97.58%)	1,012 (62.70%)	563 (34.88%)	15 (0.93%)	24 (1.49%)
**2**	1,574 (97.52%)	1,020 (63.20%)	554 (34.42%)	12 (0.74%)	28 (1.73%)

### Mapping back Illumina short and PacBio HiFi reads

In addition, Illumina short reads were mapped back to the haplotype-resolved assemblies using BWA (v0.7.10-r789; mode: aln; default settings)^25^ to assess completeness and read distribution. The mapping ratio exceeded 99.3% for both haplotypes, and approximately 83.5% of reads were properly mapped, i.e., paired clean reads that mapped to the same reference sequence within a defined distance threshold (Table 10), confirming the high assembly integrity. Sequencing depth and coverage analysis revealed an average depth of 114–116 × , with >99.3% of the genome covered at ≥20× (Table 11). These metrics indicate uniform sequence representation and negligible missing regions, demonstrating that the assembled genomes are highly complete and suitable for downstream analyses.

**Table 10:** Statistics of Illumina short read mapping.

Haplotype	Total reads (M)	Mapped reads	Properly mapped reads
1	535.7 M	532.3 M (99.4%)	447.4 M (83.5%)
2	535.7 M	532.2 M (99.4%)	447.3 M (83.5%)

**Table 11:** Sequencing depth and coverage of Illumina short reads.

Haplotype	Average sequencing depth	Coverage	Coverage (≥5×)	Coverage (≥10×)	Coverage (≥20×)
1	114×	99.7%	99.6%	99.5%	99.3%
2	116×	99.8%	99.7%	99.7%	99.6%

PacBio HiFi reads were also mapped back to the haplotype assemblies using Minimap2^74^, yielding similar results, with mapping ratios above 99.7% for both haplotypes, average sequencing depths of 154–155×, and ≥20× genome coverage for >99.1% of the genome.

### k-mer-based evaluation with Merqury

The quality and completeness of the haplotype assemblies were further assessed using a k-mer-based approach with Merqury^75^. HiFi raw reads were first adapter-trimmed using Cutadapt^76^ (Galaxy v5.1 + galaxy0^69^), and the trimmed reads were then processed with Meryl^75^ (Galaxy v1.3 + galaxy6^69^) to generate 31-mer counts. The individual k-mer databases were merged using the union-sum method to create a comprehensive reference k-mer database representing the full diploid sequence content. Merqury^75^ (Galaxy v1.3 + galaxy4^69^) was then used to evaluate how many of the raw-read k-mers were present in each assembly, enabling an independent assessment of completeness for haplotype 1, haplotype 2, and the combined diploid assembly. The Merqury analysis demonstrated a high level of completeness for both haplotype assemblies: 77.2% of the expected 578.5 M k-mers were recovered in haplotype 1 and 76.9% in haplotype 2. The combined diploid assembly recovered 99.5% of the k-mers, demonstrating that the two haplotypes together capture nearly the entire diploid genome with high completeness.

### LTR Assembly Index

The LTR Assembly Index (LAI)^77^ was calculated separately for each haplotype to assess assembly continuity within long terminal repeat (LTR) retrotransposon-rich regions. For each haplotype, the assembly was first indexed using the GenomeTools suffixerator (GenomeTools v1.6.2)^78^. LTR retrotransposon candidates were then identified with LTRharvest^32^ (GenomeTools v1.6.2)^78^ and subsequently curated and filtered using LTR_retriever v2.9.0^31^. Haplotype 1 yielded a genome-wide LAI of LAI₁ = 11.4, while haplotype 2 showed a comparable value of LAI₂ = 12.6. With LAI values ≥ 10, both assemblies meet the criteria for reference-quality genomes^77^, indicating that LTR retrotransposons are reconstructed with high continuity across both haplotypes.

### Application of the genome assembly in fire blight resistance mapping

**Fig. 5:**
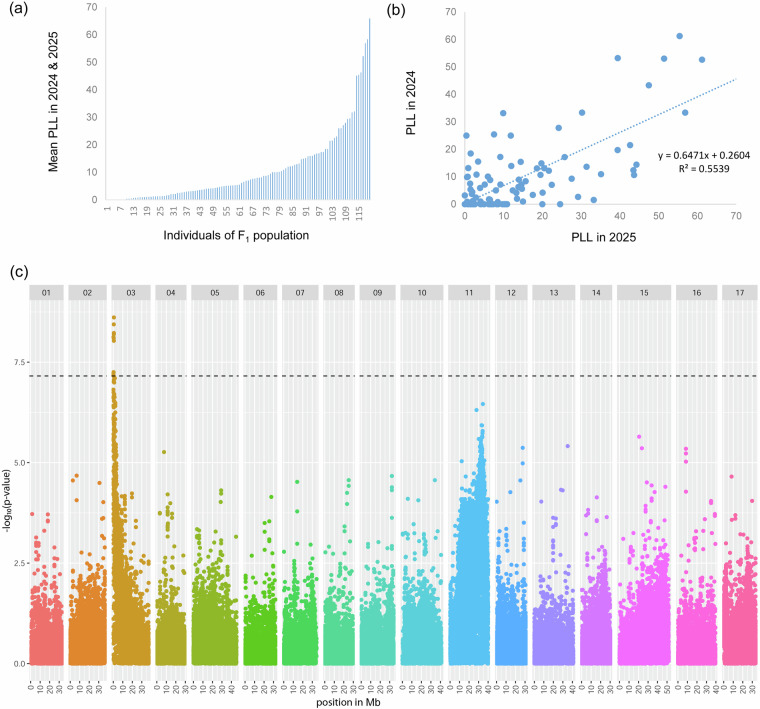
PLL in the F_1_ biparental (‘Idared’ × *M. baccata* ‘Jackii’) population after artificial fire blight inoculation. (**a**) Mean necrosis (%) across 2024 and 2025. (**b**) Correlation of necrosis between years across F_1_ genotypes. (**c**) Manhattan plot of the genome-wide association for mean PLL in the F_1_ population with newly generated SNP markers of haplotype 2. The dashed line indicates the Bonferroni-corrected significance threshold. Mb: million base pairs, PLL: percent lesion length, SNP: single-nucleotide polymorphism.

To demonstrate the usability and quality of the genome assembly for downstream applications, we applied it in an association mapping approach to identify the fire blight resistance locus of *M. baccata* ‘Jackii’, which had been previously proposed^17^. A total of 119 progenies derived from an ‘Idared’ × *M. baccata* ‘Jackii’ cross, as well as both parental genotypes, were grafted onto rootstock MM111, and up to five replicates per genotype were phenotyped for fire blight incidence i.e., length of shoot tip necrosis after artificial inoculation using *E. amylovora* strain *Ea*222, as described in Peil *et al*.^14^. Mean percent lesion length (PLL) was calculated for each F_1_ genotype by dividing the length of necrotic shoot by the total shoot length and averaging data from experiments conducted in 2024 and 2025. To identify potential associations between SNPs and fire blight incidence, each SNP from the aforementioned tGBS analysis with a minor allele frequency (MAF) ≥ 0.05 was analysed using the following procedure: genotypes were divided into two groups according to the observed allele. Using ‘Idared’ as a reference for the susceptible allele, phenotypic values of the two groups were used for a non-parametric Wilcoxon rank-sum test performed in R v4.4.2^79^. The genome-wide significance threshold was determined using Bonferroni correction as -log_10_(0.01/number of SNPs tested). A Manhattan plot was generated using the ggplot2 package^80^ and it showed a significant association of SNP markers at the top of chromosome 3 with the fire blight phenotypic data of the F_1_ progeny, shown for haplotype 2 in Fig. 5. Haplotype 1 produced a comparable plot (not shown). The sequence of the *FB_Mr5* homolog in *M. baccata* ‘Jackii’ (GenBank accession KT013244.1^17^) was found to be 100% identical over 4,164 bp to a region at the top of chromosome 3 of haplotype 2 between positions 598,337 and 602,501 bp, and this homolog is only 43,365 bp distant from the SNP marker with the highest -log₁₀(p) value and the strongest association with fire blight resistance. This supports the accuracy of the haplotype-resolved genome assembly, the correctness of chromosome assignment and orientation, as *FB_Mr5* has been described to be located on the distal part of chromosome 3^14^, and the reliability and usability of the genomic data presented here for further analyses.

## Supplementary information

quant_function_0_100

run_analysis_0_100_with filter_R1

## Data Availability

Data supporting the findings of this study are publicly available at the European Nucleotide Archive (ENA)^71^ and Figshare^72^.

## Code availability

A custom R script was used to perform association mapping and is provided as supplementary information.
