# Supplementary material for: High-quality haplotype-resolved genome assembly and annotation of Malus baccata ‘Jackii’
Source: Sci Data. 2026 Jan 8;13:14. doi: 10.1038/s41597-025-06504-5 (PMC12783112; doi:10.1038/s41597-025-06504-5)
Supplement: Supplementary file 1 — quant_function_0_100 [file 41597_2025_6504_MOESM1_ESM.pdf]

```
##read VCF
readVCF<-function( geno ) {
	#determine number of comment lines
	con = file(geno, "r")
	skip=0;
	while ( TRUE ) {
		line = readLines(con, n = 1)
		if ( !startsWith(line,"##") ) {
			break
		}
		#print(line)
		skip=skip+1;
	}
	close(con)

	#read vcf
	d=as.matrix(read.delim(geno,skip=skip,sep="\t",check.name
s=F))
	return(d);
}

##create 0-1-2-matrix with alternative allele counts
createAlternativeAlleleCountMatrix <- function( vcf ) {
	t(apply(vcf, 1, function(y) {
		if (y[9] == "GT") {
			x = y[-(1:9)]
		} else {
			idx = which(strsplit(y[9], ":")[[1]] == "GT")
			x = sapply(y[-(1:9)], function(z)  strsplit(z,
":")[[1]][idx])
		}

		z = rep(NA, length(x))
		z[x %in% c("0/0", "0|0")] = 0
		z[x %in% c("0/1", "0|1", "1/0", "1|0")] = 1
		z[x %in% c("1/1", "1|1")] = 2
		names(z) = names(x)
		return(z)
	} ))
}

##compute p values
associateQuantTrait <- function(chr, pos, aacm, quantAll,
susceptible, resistant, alternative = "two.sided") {
	qNames = intersect(colnames(aacm), names(quantAll))
	quant  = quantAll[qNames]
	names  = unique(c(names(quant), susceptible, resistant))

	pVal = t(apply(aacm, 1, function(y) {
		zz = y[qNames]


		if (is.na(y[susceptible]) || is.na(y[resistant]) ||
y[susceptible] == 1 || y[resistant] == y[susceptible]) {
			res = rep(NA, 4)
		} else {
			if (y[susceptible] == 0) {
```

```
                        idx1 = which(zz == 0)
                        idx2 = which(zz > 0)
                  } else { #if(y[suceptible] == 2) {
                        idx1 = which(zz == 2)
                        idx2 = which(zz < 2)
                  }
                  h = !is.na(zz); #wichtig, das sonnst MAF
falsch berechnet wird
                  af = sum(zz[h])/(2*length(zz[h]))
                  res = c(length(idx1), length(idx2), NA,
min(af, 1 - af))

                  if (min(res[1:2]) > 10) {
                        res[3] = wilcox.test(quant[idx1],
quant[idx2], alternative = alternative)$p.value
                  }
            }

            return(res)
      }))

      colnames(pVal) = c("group1", "group2", "p-Value", "MAF")
      idx = which(!is.na(pVal[, 1]))

      return(data.frame(
           Index      = idx,
           Chromosome = chr[idx],
           Position   = as.numeric(pos[idx]) / 1E6,
           Group1     = pVal[idx, 1],
           Group2     = pVal[idx, 2],
           LOD        = -log10(pVal[idx, 3]),
           MAF        = pVal[idx, 4]
        ))
}

require(ggplot2)

##draw manhattan
manhattan <- function( results, name=NA, signif=0.01,
common=NA ) {
      df = results
      if( !is.na(common) ) {

      df[,"Chromosome"]=gsub(common,"",df[,"Chromosome"]);
      }
      s=-log10( signif / nrow(df) )
      g = ggplot( df, aes(x=Position, y=LOD, col=Chromosome,
ymin=0) ) +
            facet_grid( ~Chromosome, scales="free_x",
space="free_x" ) +
            theme(legend.position="none") +
            geom_point() +
            geom_hline( yintercept=s, linetype=2 ) +
            xlab("Position in Mb") +
            theme(axis.text.x = element_text(angle = 90, vjust
= 0.5, hjust=1));
      if( !is.na(name) ) {
```

```
            g = g + ggtitle(name)
      }
      return(g);
}

#draw phenotype per alternative allele count
getPeak <- function( trait, chr, pos, z, quantAll ) {
      qNames=intersect(names(z),names(quantAll))
      df=data.frame(sample=qNames, quant=quantAll[qNames],
alternativeAlleleCount=z[qNames]);

      g = ggplot(df, aes(x = quant)) +
        facet_grid(~alternativeAlleleCount) +
        ggtitle(paste(trait, ": ", chr, " @ ", pos,sep="")) +
        geom_histogram(binwidth = 5, color = "black", fill =
"steelblue") +
        scale_x_continuous(limits = c(0, 100), breaks = seq(0,
100, by = 10))

      return( list( plot=g, data=df) );
}
```
