# Supplementary material for: High-quality haplotype-resolved genome assembly and annotation of Malus baccata ‘Jackii’
Source: Sci Data. 2026 Jan 8;13:14. doi: 10.1038/s41597-025-06504-5 (PMC12783112; doi:10.1038/s41597-025-06504-5)
Supplement: Supplementary file 2 — run_analysis_0_100_with filter_R1 [file 41597_2025_6504_MOESM2_ESM.pdf]

```
# ------------------------------------------
# Quantitative association with VCF data
# ------------------------------------------

setwd("D:/projects/Apfel/neu/")

# --- Load custom functions ---
source("quant_function_0_100.R")  # contains readVCF(),
associateQuantTrait()

# --- Set parameters ---
genoFile     = "Galaxy33-
[adjusted_Apple.Chr.HT1.Analysis.MCR50.snps.chrOnly.imputed.vc
f].vcf"
quantFile    = "quant_trait_2_without.tsv"
susceptible  = "Idared"
resistant    = "Jackii"

# --- Read VCF ---
vcf = readVCF(genoFile)

removeSamples <- c("05225_27", "05225_30", "06228_5", "06228_
6", "06228_54",
                   "06228_73", "06228_91", "06228_108")   #
can be extended, e.g. c("05225_27","06228_41")

# --- Shorten sample names in VCF (from column 10 onward) ---
vcfSamples     = colnames(vcf)[10:ncol(vcf)]
shortNames     = sub("\\..*", "", vcfSamples)  # remove
everything after the first dot
colnames(vcf)[10:ncol(vcf)] = shortNames

# --- Read trait file (with NA detection enabled!) ---
quantAll = read.delim(quantFile, sep = "\t", header = TRUE,
check.names = FALSE,
                      row.names = 1, na.strings = "NA")

# --- Analysis ---
aacm = createAlternativeAlleleCountMatrix(vcf)
pdf(paste("manhattan_plot-", genoFile, ".pdf", sep=""), width
= 12)

t(sapply(1:ncol(quantAll), function(i) {
    trait    = colnames(quantAll)[i]
    q        = quantAll[, i]
    names(q) = rownames(quantAll)

    # --- Convert trait values to numeric (with warning
suppression) ---
    q = suppressWarnings(as.numeric(q))
    names(q) = rownames(quantAll)

    # --- Use only valid (non-NA) values ---
    q = q[!is.na(q)]

    # --- Restrict VCF to shared samples ---
    common = intersect(colnames(aacm), names(q))
```

```
    m      = aacm[, common]
    q      = q[common]

    # --- Perform association analysis ---
    results = associateQuantTrait(vcf[,1], vcf[,2], m, q,
                                  susceptible = susceptible,
                                  resistant = resistant)

    # --- Save results ---
    write.table(results,
                paste("quant_results-", genoFile, "-", trait,
".tsv", sep=""),
                sep = "\t", quote = FALSE, col.names = NA)

    # --- Determine common prefix ---
    u = unique(vcf[, 1])
    for(i in 2:length(u)) {
        p = 1
        while(substr(u[1], p, p+1) == substr(u[i], p, p+1)) {
            p = p + 1
        }
    }
    common = substr(u[1], 1, p)

    # --- Generate Manhattan plot ---
    g = manhattan(results, trait, common = common)
    print(g)

    # --- Peak plot for best SNP result ---
    bestIdx        = which.max(results[, "LOD"])      # index
of top SNP
    vcf_row_index  = results[bestIdx, "Index"]        # actual
row index in VCF
    peak = getPeak(trait, vcf[vcf_row_index,1],
vcf[vcf_row_index,2],
                   m[vcf_row_index,], q)
    print(peak[["plot"]])

    return(cbind(trait, vcf[vcf_row_index,1],
vcf[vcf_row_index,2]))
}))
dev.off()

## Just for demonstration

# --- Minor allele frequency ---
maf = apply(aacm, 1, function(x) {
    r = sum(x, na.rm = TRUE) / (2 * length(which(!is.na(x))))
    min(r, 1 - r)
})
hist(maf, breaks = 50)
abline(v = 0.2, lty = 2, col = 2)
a = maf >= 0.05

res = associateQuantTrait(vcf[a,1], vcf[a,2], m[a,], q,
                          susceptible = susceptible,
                          resistant = resistant)
```

```
manhattan(res, trait, common = common)

# --- Test cases where only one allele is never homozygous ---

bool = t(apply(aacm, 1, function(x)
    c(length(which(x == 0)) == 0,
      length(which(x == 2)) == 0)))

c1 = a & bool[,1]
res = associateQuantTrait(vcf[c1,1], vcf[c1,2], m[c1,], q,
                          susceptible = susceptible,
                          resistant = resistant)
manhattan(res, trait, common = common)

b = apply(bool, 1, any)
c2 = a & b
res = associateQuantTrait(vcf[c2,1], vcf[c2,2], m[c2,], q,
                          susceptible = susceptible,
                          resistant = resistant)
manhattan(res, trait, common = common)
```
